# Association of body mass index and inflammatory dietary pattern with breast cancer pathologic and genomic immunophenotype in the nurses’ health study

**DOI:** 10.1186/s13058-022-01573-5

**Published:** 2022-11-14

**Authors:** Sarah Asad, Adrienne Damicis, Yujing J. Heng, Kathryn Kananen, Katharine A. Collier, Elizabeth J. Adams, Kevin H. Kensler, Gabrielle M. Baker, Robert Wesolowski, Sagar Sardesai, Margaret Gatti-Mays, Bhuvaneswari Ramaswamy, A. Heather Eliassen, Susan E. Hankinson, Fred K. Tabung, Rulla M. Tamimi, Daniel G. Stover

**Affiliations:** 1 Division of Medical Oncology, Stefanie Spielman Comprehensive Breast Center, Ohio State University Comprehensive Cancer Center, Biomedical Research Tower, Room 984, Columbus, OH 43210 USA; 2 Division of Epidemiology, College of Public Health, Ohio State University, Columbus, OH 43210 USA; 3 Department of Pathology, Beth Israel Deaconess Medical Center, Boston, MA 02215 USA; 4 Channing Division of Network Medicine, Department of Medicine, Brigham and Women’s Hospital, Harvard Medical School, Boston, MA 02115 USA; 5 Department of Medical Oncology, Dana-Farber Cancer Institute, Boston, MA 02115 USA; 6 Department of Epidemiology, Harvard T.H. Chan School of Public Health, Boston, MA 02115 USA; 7 Ohio State University College of Medicine, Columbus, OH 43210 USA; 8 Department of Biostatistics and Epidemiology, University of Massachusetts School of Public Health and Health Sciences, Amherst, MA 01003 USA; 9 Department of Population Health Sciences, Weill Cornell Medicine, New York, NY 10065 USA; 10 Department of Biomedical Informatics, Ohio State University, Columbus, OH 43210 USA; 11 Northwestern Feinberg School of Medicine, Chicago, IL 60611 USA

**Keywords:** Breast cancer, Tumor microenvironment, Tumor infiltrating lymphocytes, Gene expression signature

## Abstract

**Background:**

Breast tumor immune infiltration is clearly associated with improved treatment response and outcomes in breast cancer. However, modifiable patient factors associated with breast cancer immune infiltrates are poorly understood. The Nurses’ Health Study (NHS) offers a unique cohort to study immune gene expression in tumor and adjacent normal breast tissue, immune cell-specific immunohistochemistry (IHC), and patient exposures. We evaluated the association of body mass index (BMI) change since age 18, physical activity, and the empirical dietary inflammatory pattern (EDIP) score, all implicated in systemic inflammation, with immune cell-specific expression scores.

**Methods:**

This population-based, prospective observational study evaluated 882 NHS and NHSII participants diagnosed with invasive breast cancer with detailed exposure and gene expression data. Of these, 262 women (training cohort) had breast tumor IHC for four classic immune cell markers (CD8, CD4, CD20, and CD163). Four immune cell-specific scores were derived via lasso regression using 105 published immune expression signatures’ association with IHC. In the remaining 620 patient evaluation cohort, we evaluated association of each immune cell-specific score as outcomes, with BMI change since age 18, physical activity, and EDIP score as predictors, using multivariable-adjusted linear regression.

**Results:**

Among women with paired expression/IHC data from breast tumor tissue, we identified robust correlation between novel immune cell-specific expression scores and IHC. BMI change since age 18 was positively associated with CD4+ (*β* = 0.16; *p* = 0.009), and CD163 novel immune scores (*β* = 0.14; *p* = 0.04) in multivariable analyses. In other words, for each 10 unit (kg/m^2^) increase in BMI, the percentage of cells positive for CD4 and CD163 increased 1.6% and 1.4%, respectively. Neither physical activity nor EDIP was significantly associated with any immune cell-specific expression score in multivariable analyses.

**Conclusions:**

BMI change since age 18 was positively associated with novel CD4+ and CD163+ cell scores in breast cancer, supporting further study of the effect of modifiable factors like weight gain on the immune microenvironment.

**Supplementary Information:**

The online version contains supplementary material available at 10.1186/s13058-022-01573-5.

## Background

Tumor infiltrating lymphocytes (TILs) have been associated with improved response to chemotherapy and better overall survival in breast cancer [1–4], and conversely, there is evidence that suppressive immune cells facilitate tumor evasion of the host immune system [5]. More specifically, the presence of CD8+ T cells is associated with improved long-term survival in both HER2+ and triple-negative breast cancers [6], and there is evidence that chemotherapeutic agents promote CD8+ T cell-mediated immunogenic tumor cell killing [7]. Evidence suggests that tumor-specific factors, such as hormone receptor (HR) status and molecular subtype, are key associations with immune infiltration in breast tumors [6]. However, these factors do not fully describe the observed variation in the tumor immune microenvironment of breast cancers and there is growing evidence that patient-level modifiable factors may also play a role.

Recent studies show that body mass index (BMI) is significantly correlated with systemic inflammation [8] and may influence inflammation within the breast microenvironment. BMI has been shown to be positively correlated with breast tumor and normal tissue inflammation in patients with ER-negative breast cancer [9]. Interestingly, overweight or obese patients may have improved responses to immunotherapy in other cancers [10], which could indicate a distinct tumor immune microenvironment.

There is also evidence that the inflammatory potential of an individual’s diet may influence breast cancer risk and outcomes, based on analyses of the Women’s Health Initiative cohort [11, 12]. These studies utilized a literature-derived nutrient-based dietary inflammatory index to assess the inflammatory potential of the diet. A related inflammatory index, the empirical dietary inflammatory pattern (EDIP), is a food-based score of dietary inflammatory potential [13, 14] that has been shown to be associated with the immune microenvironment in colorectal cancer. Specifically, pro-inflammatory diet (higher EDIP score) was associated with colorectal cancers low or no intratumoral periglandular reaction (fewer/no immune cells), but not with cancers that had intermediate or high peritumoral lymphocytic reaction [15].

Based on these data, we hypothesized that patient factors including adiposity (BMI change from age 18), physical activity, and dietary inflammatory potential (EDIP score) may play an important role in the breast cancer tumor immune microenvironment. In the current study, we aimed to develop immune cell-specific expression signatures based on immunohistochemistry (IHC) data, and then, we evaluated associations of BMI change since age 18, physical activity, and EDIP score with the expression signatures.

## Methods

### Study population

The Nurses’ Health Study (NHS) was established in 1976 with the enrollment of 121,700 US female registered nurses aged 30–55 years, while the Nurses’ Health Study II (NHSII) consists of 116,429 US female registered nurses aged 25–42 years who were enrolled starting in 1989. Detailed descriptions of cohort procedures have been reported elsewhere [16]. Briefly, cohort members completed baseline questionnaires that provided medical histories and extensive information about demographic, lifestyle, reproductive, and dietary risk factors for breast cancer. Both cohorts have been followed biennially by mailed questionnaire to update information on exposure status and ascertain newly diagnosed diseases, including cancers. All women reporting incident diagnoses of breast cancer were asked for permission to review their medical records; 99% of reported cases were confirmed with pathology reports and medical record review. Tumor characteristics were extracted from pathology reports. Immunohistochemical evaluation of estrogen receptor (ER), progesterone receptor (PR), and human epidermal growth factor receptor 2 (HER2) expression was obtained from central review of breast tissue microarrays [16]. We analyzed 882 participants enrolled in the NHS and NHSII and diagnosed with invasive breast cancer, who had detailed exposure and follow-up data and gene expression data [9, 17]. The study protocol was approved by the institutional review boards of the Brigham and Women’s Hospital and Harvard T.H. Chan School of Public Health, and those of participating registries as required.

### Empirical dietary inflammatory pattern (EDIP) score

The empirical dietary inflammatory pattern (EDIP) score is a food-based dietary index for assessing dietary inflammatory potential, and it is based on circulating concentrations of C-reactive protein, interleukin-6, and tumor necrosis factor alpha receptor 2. EDIP is a weighted sum of 18 food groups from food frequency questionnaires (FFQs) derived in the NHS [14] and validated in NHSII and Health Professionals Follow-Up Study [13, 14]. The component food groups comprising the EDIP score that are positively associated with concentrations of inflammatory markers are: processed meat, red meat, organ meat, non-dark fish, vegetables other than green leafy vegetables/dark yellow vegetables, refined grains, high energy beverages (carbonated beverages with sugar, fruit drinks), low-energy beverages (low-energy cola/other carbonated beverages), and tomatoes. The component food groups that are inversely associated with concentrations of inflammatory markers are: beer, wine, tea, coffee, dark yellow vegetables (carrots, yellow squash, and sweet potatoes), green leafy vegetables, snacks, fruit juice, and pizza [14]. As previously described, the EDIP score in NHS/NSII ranges from − 3.34 to 2.81, with higher scores associated with higher concentrations of inflammatory markers [14]. Cumulative average EDIP uses all prior available FFQs (median 8, range 5–8 FFQs).

### Body mass index and other covariates

Weight and height were reported on study questionnaires, and self-reported weight and height were previously validated in the NHSII [18]. Height was obtained at enrollment, while weight (and other covariates) was updated every 2 years, starting in 1976 (NHS) or 1991 (NHSII). BMI (kg/m^2^) was calculated using weight from the participant’s last available questionnaire before breast cancer diagnosis (i.e., within 2–4 years of diagnosis). BMI change since age 18 was calculated as the last available pre-diagnosis BMI minus BMI at age 18. BMI change since age 18 provides a potential surrogate for adiposity relative to BMI alone, as significant rise in BMI from age 18 typically reflects increase in non-lean body mass [19]. Prior studies suggest adult weight gain captures dynamic pattern of weight trajectory throughout adult life reflecting a time-integrated metric, adults gain weight mostly through accumulating fat mass and detrimental fat distribution, and adult weight gain is a simpler and more intuitive concept to the general public than BMI [19]. As a sensitivity analysis, the last available pre-diagnosis BMI was converted to a categorical variable with three levels (underweight/normal weight, overweight, and obese) as determined by the standard BMI thresholds for women (< 25, 25–29.9, > 30). Very few participants had a BMI category of “underweight” (*N* = 11/882, 1.2%) so the “underweight” and “normal weight” categories were combined for this analysis. Further sensitivity analyses assessed pre-diagnosis BMI as a continuous predictor and BMI at age 18 as a continuous predictor. For physical activity, we used cumulative average metabolic equivalent task (MET) hours per week to capture long-term activity. Other covariates such as race, age at first birth, parity, age at diagnosis, year of diagnosis, menopausal status, recent postmenopausal hormone therapy (HT), and smoking [20] were retrieved from baseline, subsequent, or most recent NHS/NHSII questionnaires. When available, the most recent pre-diagnosis questionnaire was prioritized.

### Tissue microarray and immunohistochemistry

Tumor microarrays (TMA) were constructed from archived formalin-fixed paraffin-embedded (FFPE) breast tumor blocks at the Specialized Histopathology Core of the Dana Farber/Harvard Cancer Center with three 0.6 mm core biopsies taken from the tissue blocks for each tumor [16]. IHC was conducted on 5 μm paraffin sections of the TMA blocks for CD8 (Dako 7103, clone C8/144B), CD4 (Dako 7310, clone 4B12), CD20 (Dako 0755, clone L26), and CD163 (Vector Labs VP-C374, clone 10D6) [21]. Staining positivity was determined using an automated computational image analysis system (Definiens Tissue Studio software, Munich, Germany). The percent of positive cells for each antibody was determined as the number of cells positive over the total number of cells (nuclei) for each of the three cores from each patient. The final mean percent positivity was the average of the three cores.

### Gene expression microarray, RNA sequencing, and quality control analysis

For NHS, RNA was extracted from three cores of 1 mm or 1.5 mm diameter taken from FFPE blocks of tumor tissue as described previously [22]. RNA expression was determined using Affymetrix Glue Grant Human Transcriptome Array [23]. Gene expression data were normalized and summarized using robust multiarray average (Affymetrix Power Tools v1.18.0), correcting for batch effects using a surrogate variable analysis R package, ComBat, and excluding low expressing genes (< 25th percentile) [9, 17]. The final dataset consisted of 15,369 annotated probesets of coding and non-coding RNAs. All microarray and annotation data are available at the National Center for Biotechnology Information Gene Expression Omnibus (accession number: GSE115577). For The Cancer Genome Atlas (TCGA) [24], gene expression data were obtained from XENAbrowser (version 2015-02-24) for breast cancer samples with paired tumor and normal expression data.

### Published immune gene expression signature scores

Published immune signature scores were derived as the median value of the genes in each gene set from gene expression data for each patient’s tumor, a validated approach for analyses of disparately derived signatures [25]. This included 9 gene expression signatures demonstrated to correlate with infiltrating immune cells [26–30], including the GeparSixto (GSAct) 6-gene signature (*CXCL9*, *CCL5*, *CD8A*, *CD80*, *CXCL13*, *CR2*), which was derived based on association with tumor infiltrating lymphocytes in the neoadjuvant chemotherapy breast cancer clinical trial GeparSixto [26]. In addition, we calculated the immune score for each of the 96 gene sets in the Immune Response In Silico (IRIS) signatures [31], a compendium of immune cell-specific gene sets derived to reflect immune cell activation/exhaustion states, available in ImmuneSigDB [32].

### CD4+, CD8+, CD20+, and CD163+ immune gene expression score generation

**Fig. 1:**
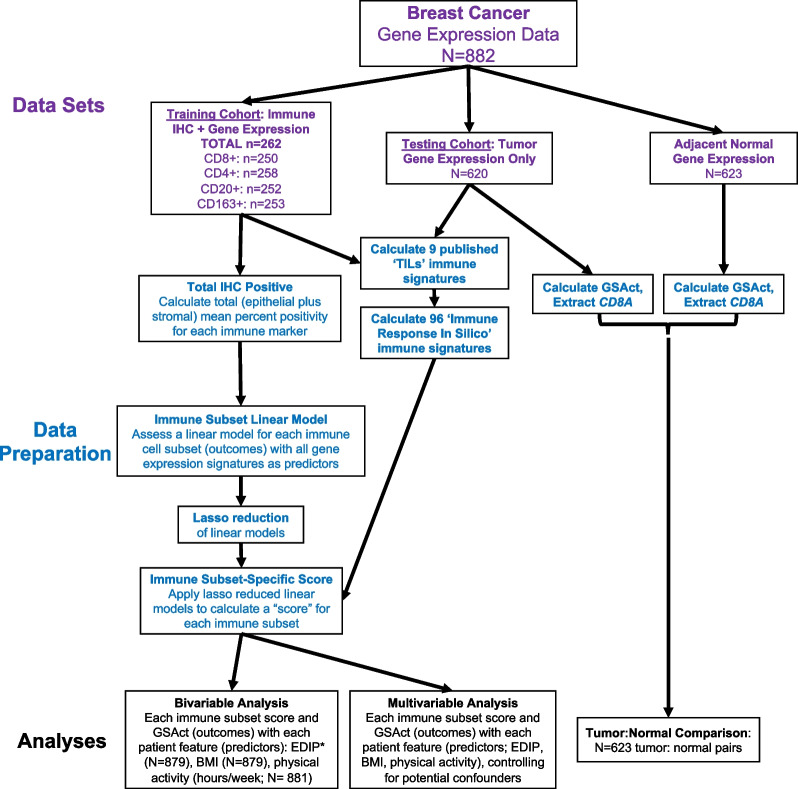
Analysis schema. Study analysis schema visualized. Datasets (purple text) indicated at top. Data preparation (blue text) included gene expression extraction, expression signature calculation, immune immunohistochemistry (IHC) quantification, as well as immune subset linear model, lasso reduction, and score calculation. Analyses (black text) included tumor/normal comparison of GSAct and *CD8A* single gene as well as bivariable and multivariable analyses of immune scores with patient features of interest: empiric dietary inflammatory potential (EDIP) score as continuous variable, body mass index (BMI) as normal/overweight/obese, and physical activity

For each of the four immune IHC markers of interest (CD4, CD8, CD20, and CD163), we calculated total mean percent positivity as described above. As none of the published immune signature scores individually demonstrated robust association with IHC, we evaluated linear models (four models, one for each immune cell subset) composed of all 105 published immune signatures available as predictors and the mean percent IHC positivity for each subset as the outcome. We then performed lasso reduction on these models, resulting in linear models of signatures that are significantly associated with the mean percent of IHC positive immune cells for each subset. We used the models to calculate an immune cell-specific score for each patient’s tumor, derived as a weighted arithmetic sum of the linear model for each patient’s gene expression data. The predicted scores represent the predicted amount of that particular IHC marker, with higher score corresponding to higher IHC. These scores were the main outcomes of interest for the present analyses (analyses schema in Fig. 1).

### Statistical analysis

We evaluated four immune scores based on the lasso reduction of IRIS gene expression signatures that were associated with IHC for four immune cell-specific markers: CD4+, CD8+, CD20+, and CD163+. We assessed the correlation of each of the immune scores with the other three scores. As an established comparison, we also assessed the GSAct. Correlations were measured using Spearman rho correlation coefficients and corresponding p values. Bivariable and multivariable linear models were used to compare the CD4+, CD8+, CD20+, and CD163+ immune scores (outcomes) with BMI change since age 18, physical activity, and EDIP score (predictors). Covariates included in the multivariable analyses are detailed above. All analyses and figure generation were performed using R version 3.5.1.

## Results

### Cohort characteristics

**Fig. 2:**
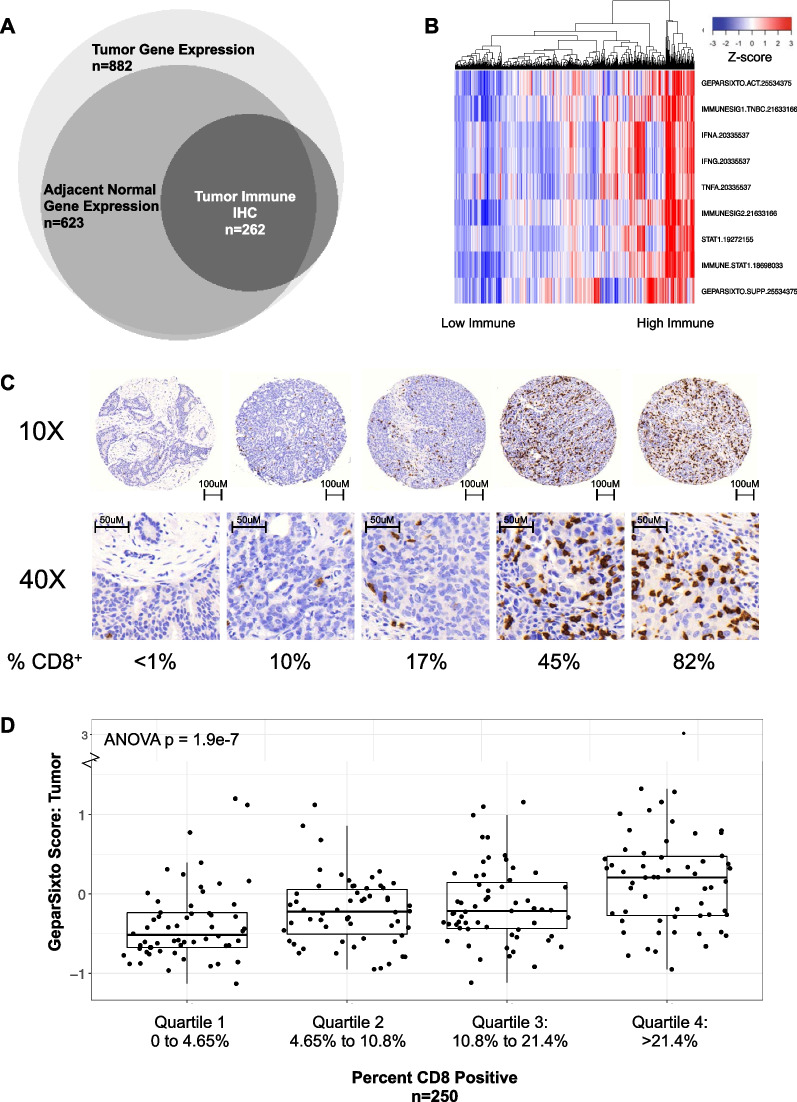
Breast cancer tumor/immune microenvironment in Nurses’ Health Study cohort. **A** Visualization of number and overlap of available data types among 882 patients in NHS/NHSII with tumor gene expression. **B** Nine published immune gene expression signatures shown to correlate with infiltrating immune cells were calculated as per primary reference; number indicated for each signature references PubMed ID. Each score was converted to Z-score across all patients in NHS gene expression cohort, with higher Z-score indicating higher immune expression score. Samples underwent unsupervised hierarchical clustering by sample. **C** Example immunohistochemistry (IHC) images from five representative tumor cores, with 10X (top) and 40X (bottom) magnification, scale bars indicated. Computational determination of CD8 positive percentages indicated below IHC images. **D** GeparSixto activation (GSAct) immune signature score (y-axis) versus CD8 quantification by quartile (x-axis), with percentage CD8 cells indicated

A total of 882 subjects enrolled in NHS or NHSII with available gene expression data derived from tumor samples were included in the study (Table 1; Fig. 2A). Overall, most patients in the cohort were white (95.5%; 842/882), stage I–II at diagnosis (90.6%; 799/882), and the majority of patients had ER+/HER2−negative breast cancer, followed by ER±/HER2+ and TNBC (60.2%, 27.4%, and 12.4%, respectively) (Additional file 1: Table S1). Of the gene expression cohort, 623 also had gene expression data derived from adjacent normal breast tissue and 262 subjects had IHC data for at least one immune marker (Figs. 1, 2A). Participants who had tumor gene expression data plus IHC data (the “IHC training cohort”), when compared to those with expression data alone (the “expression only evaluation cohort”), were exclusively from NHSII. Thus, based on cohort differences, the IHC training cohort participants were more likely to be diagnosed after 2000 (58.0% vs. 45.2%) and less than age 60 at diagnosis (100% vs. 31.0%) (Table 1). Compared to the expression only cohort, the IHC cohort had a greater proportion of ER+/HER2− breast cancers (68.3% vs. 50.5%) and more patients with normal BMI (57.6% vs. 46.6%). There was no significant difference in stage at diagnosis, cumulative average physical activity, or cumulative average EDIP score by cohort (Table 1).

**Table 1:** Cohort characteristics

	IHC training cohort N (%)	Expression only evaluation cohort N (%)	*P* value*
Total	262	620	
NHS cohort			< 0.0001
NHS	0 (0.0)	537 (86.6)	
NHSII	262 (100.0)	83 (13.4)	
Diagnosis year			0.001
Prior to 1990	1 (0.4)	13 (2.1)	
1990–1999	109 (41.6)	327 (52.7)	
2000–2011	152 (58.0)	280 (45.2)	
Age at diagnosis (years)			< 0.0001
< 50	156 (59.5)	29 (4.7)	
50–59	106 (40.5)	163 (26.3)	
60–69	0 (0.0)	230 (37.1)	
≥ 70	0 (0.0)	198 (31.9)	
IHC subtype			< 0.0001
ER−/HER2−	36 (13.7)	65 (10.5)	
ER+/HER2−	179 (68.3)	313 (50.5)	
ER± /HER2 +	46 (17.6)	178 (28.7)	
Race			0.20
White	246 (93.9)	596 (96.1)	
Non-White	16 (6.1)	24 (3.9)	
Menopausal status			< 0.0001
Premenopausal	200 (76.3)	37 (6.0)	
Postmenopausal	45 (17.2)	562 (90.6)	
Unknown	17 (6.5)	21 (3.4)	
Stage at diagnosis			0.83
Stage I	152 (58.0)	376 (60.6)	
Stage II	86 (32.8)	185 (29.8)	
Stage III	21 (8.0)	54 (8.7)	
Stage IV	2 (0.8)	4 (0.6)	
Body mass index (BMI)			0.01
Underweight/Normal weight (< 25)	151 (57.6)	289 (46.6)	
Overweight (25- < 30)	67 (25.6)	195 (31.5)	
Obese (≥ 30)	43 (16.4)	134 (21.6)	
BMI change since age 18			0.09
Mean	4.8	5.4	
Standard deviation	4.5	4.7	
Range	(− 4.7,22.1)	(− 11.7,24.9)	
Cumulative average physical activity (MET hours/week)			0.83
Mean	18.2	18.5	
Standard deviation	18.8	16.5	
Range	(0.7,191.3)	(0.4,117.7)	
Cumulative average empirical dietary inflammatory pattern (EDIP) score			0.15
Mean	− 0.1	− 0.04	
Standard deviation	0.8	0.8	
Range	(− 2.5,2.2)	(− 4.3,4.5)	

*P value from chi-square tests for categorical variables and t tests for continuous variables

### Characterization of established immune expression signatures in the NHS cohort

To understand the landscape of immune gene expression, in the full cohort (*n* = 882 participants) we first evaluated 105 previously published, well-established immune gene expression signatures representing general immune infiltration as well as specific immune subsets (Additional file 2: Table S2). Among these, we visualized nine general immune signatures shown to be associated with TILs [26–30] (Fig. 2B). These signatures indicated that most tumors had relatively low expression of immune-related genes, while a small subset (~ 10–15%) showed high immune gene expression.

Among tumors with paired IHC and gene expression (IHC training cohort), we evaluated the association of each of the 105 published immune signatures with IHC staining for CD8 (*n* = 250), CD4 (*n* = 258), CD20 (*n* = 252), and CD163 (*n* = 253) (Additional file 3: Table S3). Correlations were modest, with most Spearman correlation coefficients < 0.30 and none greater than 0.40. Among all signatures, the GeparSixto “immune activation signature” (GSAct), which was derived based on association with TILs in the GeparSixto clinical trial [26] and was recently confirmed to have a robust association with response to neoadjuvant chemotherapy in early-stage breast cancer [33], was among the strongest associations across all immune IHC markers (Additional file 3: Table S3). This suggested that, as designed, GSAct was a representative signature of TILs in breast tumors; thus, moving forward in analyses, we focused on GSAct. As anticipated, GSAct was highest in TNBC relative to other breast cancer receptor subtypes as well as higher in basal-like and HER2-enriched molecular intrinsic subtypes (Additional file 4: Figure S1A, B).

As an initial validation, we evaluated the association of GSAct with CD8+ IHC among 250 patients with paired CD8+ T cell immunohistochemistry and gene expression data. Overall, CD8+ quantification corresponded to visualized CD8+ cell amount (example images in Fig. 2C); this supports recent work from our group showing overall good agreement between computational evaluation of IHC with expert pathology review (Roberts, Tamimi, et al.; manuscript under review).

When stratified into quartiles by reported total CD8 IHC quantification, there was a strong association with GSAct (ANOVA *p* = 1.9e−7; Fig. 2D; CD8 IHC continuous; Additional file 4: Figure S1C-D). The overall correlation with CD8 IHC and GSAct as continuous variables remained for epithelial, stromal, and total CD8 positivity (range Pearson’s *r* = 0.45–0.47; Additional file 4: Figure S1E).

### Immune expression signatures in breast cancer and adjacent normal breast tissue

**Fig. 3:**
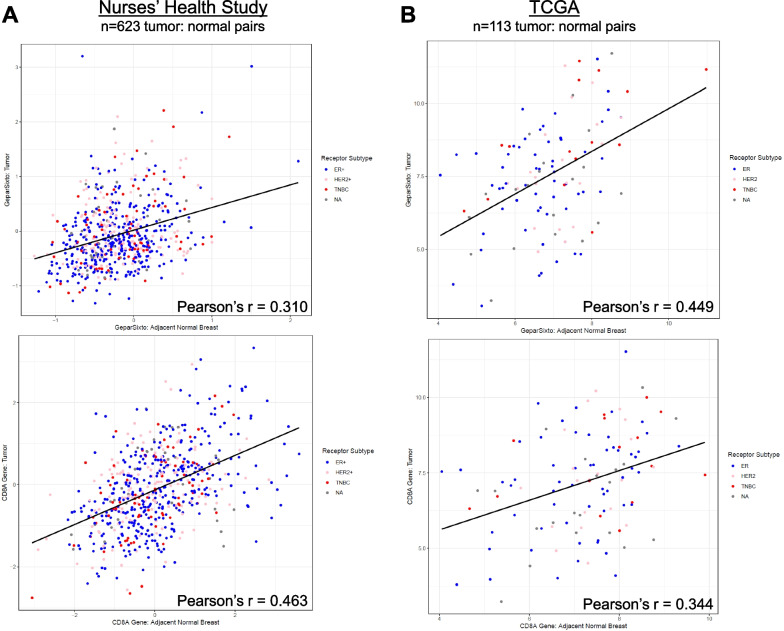
T cell immune gene expression in breast tumor and adjacent normal. GeparSixto activation (GSAct) signature was calculated as the median of 6-genes (*CXCL9*, CCL5, CD8A, CD80, CXCL13, CR2) as described previously [26, 33] for gene expression data available from tumor and adjacent normal breast in Nurses’ Health Study (**A**; top panel) and The Cancer Genome Atlas (TCGA; **B**; top panel). *CD8A* gene expression data was extracted from tumor and adjacent normal breast in Nurses’ Health Study (**A**; bottom panel) and The Cancer Genome Atlas (TCGA; **B**; bottom panel). For all panels, each dot represents a single tumor/adjacent normal pain and black line indicates line of best fit, with Pearson correlation coefficient indicated. Dot color represents receptor-based estrogen receptor positive/HER2 negative (ER, blue dots), HER2 positive (HER2, pink dots), or estrogen receptor negative/progesterone receptor negative/HER2 negative (TNBC, red dots)

The NHS cohort offers a unique opportunity to interrogate gene expression data from tumor and adjacent normal tissue. We focused our comparisons on GSAct and *CD8A* as a representative single gene for general CD8+ T cell infiltration. Among the 623 patients with paired tumor–normal samples, we observed a positive correlation between tumor and adjacent normal tissue for GSAct (Pearson’s *r* = 0.31, *p* < 0.001) and *CD8A* single gene (Pearson’s *r* = 0.46, *p* < 0.0001) (Fig. 3A). These results were validated in the independent TCGA breast cancer cohort, with 113 pairs of tumor and adjacent normal breast transcriptome data (Pearson’s *r* = 0.45, *p* < 0.0001; Pearson’s *r* = 0.34, *p* = 0.0002 for GSAct and *CD8A*, respectively; Fig. 3B). Of the adjacent normal tissue samples in NHS, 297 were classified as a PAM50 subtype other than “normal-like” [17]. As a sensitivity analysis, we removed these 297 samples, which could be a result of contamination of normal samples, and repeated all analyses (Additional file 4: Figure S2A-B), with no meaningful change in results. As an exploratory analysis, we assessed differences in paired tumor–normal samples among the NHS and TCGA cohorts using a paired Wilcoxon test. Overall, in the NHS cohort we found that GSAct and *CD8A* each differed significantly between tumor and normal samples (*p* < 0.0001 and 0.002, respectively). However, there did not appear to be a consistent trend when comparing tumor/normal by receptor subtype or PAM50 subtype for either GSAct or *CD8A* (Additional file 4: Figure S2C-F).

### Derivation of IHC-based CD4+, CD8+, CD20+, and CD163+ immune expression scores

We initially attempted to derive novel immune signatures based on individual genes’ contributions; however, we found no significant discrimination relative to published immune expression signatures (Additional file 3: Table S3). Instead, we pursued an approach using all 105 published immune gene expression signatures and lasso reduction using the IHC training cohort. Eighteen signatures remained in the CD4+ lasso regression model, 24 remained in the CD8+ model, 24 remained in the CD20+ model, and 12 remained in the CD163+ model (Additional file 1: Tables S4–7).

**Fig. 4:**
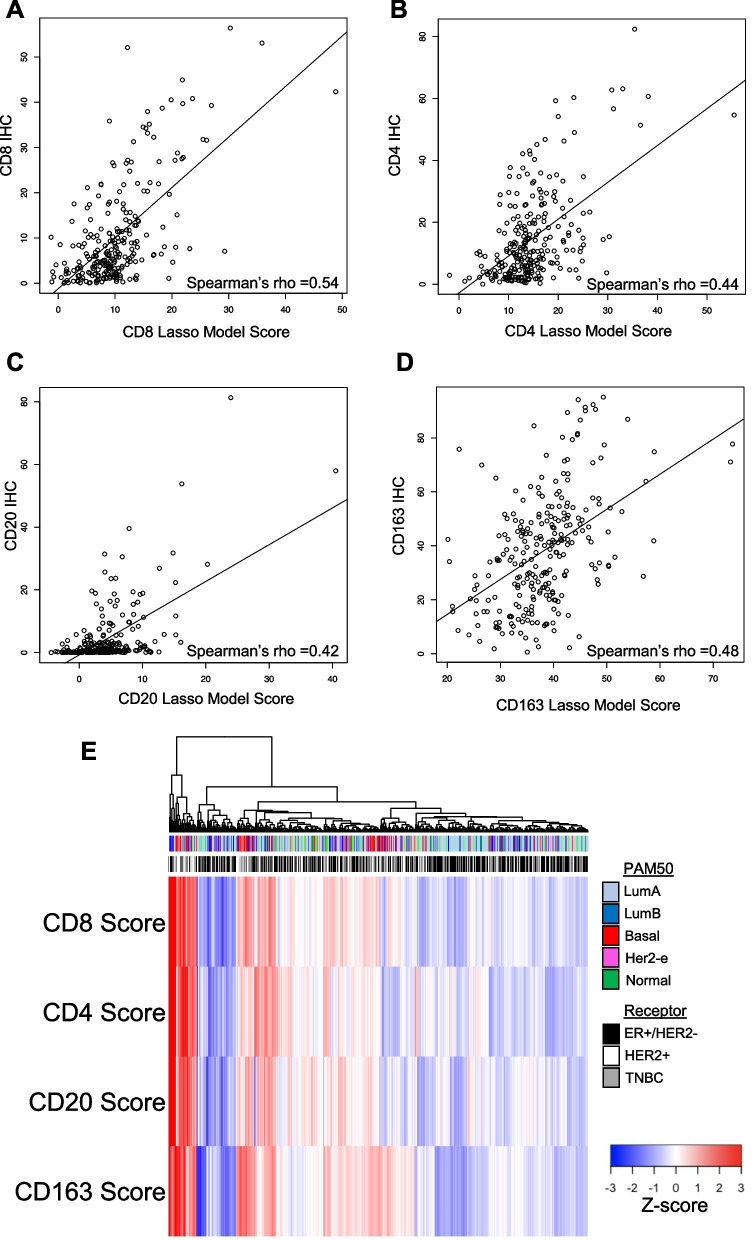
Immune cell-specific gene expression score: Association with immunohistochemistry and breast cancer subtypes. **A**–**D** Scatter plots of immunohistochemistry (IHC; y-axis) and lasso model immune cell-specific score (x-axis), with immune cell marker as indicated: CD8 (**A**); CD4 (**B**); CD20 (**C**); CD163 (**D**). Black line indicates line of best fit; Pearson’s correlation coefficient indicated. **E** Each immune cell-specific lasso model score was converted to Z-score across all patients in NHS gene expression cohort, with higher Z-score indicating higher score. Samples underwent unsupervised hierarchical clustering by sample. PAM50 intrinsic subtype and receptor subtype are indicated in color bars above

We used the final models to calculate a patient-specific score for each immune subset, derived as a weighted arithmetic sum of the linear model for each patient’s gene expression data, and plotted the score against that patient’s mean IHC percent positivity for each marker to assess the fit of our model (Fig. 4A–D). The Spearman’s rho correlation statistic ranged from 0.42 to 0.54 with *p* values all < 0.0001. We compared the performance of our four immune cell-specific lasso model scores with the 105 individual immune expression scores (9 immune signatures plus 96 IRIS gene sets; Additional file 3: Table S3). As anticipated, for each IHC marker our novel immune scores demonstrated the strongest correlation with IHC quantification. To gain a descriptive understanding, we visualized all 882 tumors using our four novel IHC-based immune expression scores along with receptor and PAM50 subtype (Fig. 4E) and assessed the correlation between each immune score with each other (Additional file 4: Figure S3A). All immune scores were significantly, positively correlated with each other (Spearman’s rho ranging from 0.57 to 0.81). CD8+ with CD20+ scores had the highest correlation (Pearson’s *r* = 0.81, *p* < 0.0001). We further assessed these correlations by IHC subtype and found similar correlations (Additional file 4: Figure S3B-D). Similarly, CD8+ and CD20+ scores were most highly correlated with each other among all IHC subtypes (Spearman’s rho: 0.79 to 0.84, all *p* < 0.0001).

### Associations of modifiable lifestyle factors with immune cell scores

Using these novel IHC-validated immune expression scores, we evaluated the association with the immune microenvironment and patient features – BMI change since age 18, physical activity, and EDIP score in the expression-only evaluation cohort (*n* = 620 participants), omitting the IHC-only cohort as the dataset on which the signatures were derived. In bivariable and multivariable analyses, there was no significant association between cumulative average physical activity (MET hours/week) and GSAct, CD8+ score, CD4+ score, CD20+ score, or CD163+ score (Tables 2, 3). Among the IHC training cohort, there was no association between BMI change since age 18 and any immune score. Among the expression only evaluation cohort, CD4+ score, and CD163+ score were positively associated with BMI change since age 18 in bivariable (*p* = 0.003, and *p* = 0.04, respectively) and multivariable (*p* = 0.009, and *p* = 0.04, respectively) analyses. In other words, for each 10 unit (kg/m^2^) increase in BMI, the percentage of cells positive for CD4 and CD163 increased 1.6% and 1.4%, respectively. In the multivariable analysis only, BMI change since age 18 was positively associated with CD8+ score (*p* = 0.05). Cumulative average EDIP score was not significantly associated with CD8+, CD4+, CD20+, or CD163+ scores in either bivariable or multivariable analyses among either cohort.

**Table 2:** Bivariable associations of lifestyle factors and immune cell signatures

Predictor	IHC cohort (*N* = 262)			Expression only cohort (*N* = 620)		
	Beta coefficient	Standard error	*P* value	Beta coefficient	Standard error	*P* value
*GeparSixto*						
BMI change since age 18	0.01	0.01	0.29	0.01	0.01	0.05
Cumulative average physical activity (MET h/week)	0.0001	0.002	0.98	0.0003	0.001	0.86
EDIP score^a^	0.08	0.05	0.10	0.04	0.03	0.15
*CD8 + score*						
BMI change since age 18	0.04	0.09	0.69	0.10	0.06	0.08
Cumulative average physical activity (MET h/week)	0.01	0.02	0.50	0.01	0.02	0.71
EDIP score^a^	0.79	0.50	0.12	0.08	0.31	0.79
*CD4 + score*						
BMI change since age 18	0.16	0.09	0.07	**0.17**	**0.06**	**0.003**
Cumulative average physical activity (MET h/week)	0.01	0.02	0.51	0.003	0.02	0.85
EDIP score^a^	0.47	0.52	0.37	0.06	0.32	0.85
*CD20 + score*						
BMI change since age 18	− 0.01	0.07	0.85	0.03	0.04	0.54
Cumulative average physical activity (MET hours/week)	0.01	0.02	0.39	− 0.002	0.01	0.85
EDIP score^a^	0.66	0.37	0.08	− 0.03	0.24	0.91
*CD163 + score*						
BMI change since age 18	0.12	0.11	0.27	**0.13**	**0.06**	**0.04**
Cumulative average physical activity (MET hours/week)	− 0.001	0.02	0.98	0.01	0.02	0.71
EDIP score^a^	0.62	0.61	0.31	0.18	0.35	0.61

Bold indicates significant values for expression only cohort

^a^Cumulative average empirical dietary inflammatory pattern (EDIP) score

**Table 3:** Multivariable-adjusted associations of lifestyle factors and immune cell signatures

Predictor		IHC cohort (*N* = 262)			Expression only cohort (*N* = 620)		
		Beta coefficient	Standard error	*P* value	Beta coefficient	Standard error	*P* value
*GeparSixto*							
BMI change since age 18		0.01	0.01	0.17	0.01	0.01	0.08
Cumulative average physical activity (MET hours/week)		0.001	0.002	0.75	0.001	0.002	0.52
EDIP score^a^		0.06	0.05	0.20	0.03	0.03	0.34
*CD8 + score*							
BMI change since age 18		0.07	0.09	0.46	0.12	0.06	0.05
Cumulative average physical activity (MET hours/week)		0.02	0.02	0.35	0.01	0.02	0.53
EDIP score^a^		0.81	0.51	0.11	− 0.08	0.34	0.81
*CD4 + score*							
BMI change since age 18		0.13	0.10	0.17	**0.16**	**0.06**	**0.009**
Cumulative average physical activity (MET hours/week)		0.03	0.02	0.24	0.01	0.02	0.54
EDIP score^a^		0.58	0.52	0.27	− 0.20	0.35	0.57
*CD20 + score*							
BMI change since age 18		0.01	0.07	0.88	0.05	0.05	0.30
Cumulative average physical activity (MET hours/week)		0.02	0.02	0.24	− 0.001	0.01	0.95
EDIP score^a^		0.71	0.38	0.06	− 0.08	0.03	0.76
*CD163 + score*							
BMI change since age 18		0.10	0.11	0.35	**0.14**	**0.07**	**0.04**
Cumulative average physical activity (MET hours/week)		0.01	0.02	0.60	0.02	0.02	0.33
EDIP score^a^		0.54	0.60	0.37	− 0.04	0.37	0.90

Multivariable linear regression models were controlled for IHC subtype, race, stage at diagnosis, year at diagnosis, age at diagnosis, NHS Cohort, and menopausal status and BMI, EDIP, and physical activity were mutually adjusted for

Bold indicates significant values for expression only cohort

^a^Cumulative average empirical dietary inflammatory pattern (EDIP) score

We performed sensitivity analyses of pre-diagnosis BMI (one two-year follow-up cycle prior to diagnosis) as a categorical predictor, pre-diagnosis BMI as a continuous predictor, and BMI at age 18 as a continuous predictor (Additional file 1: Tables S8–10). In multivariable models, categorical BMI was not associated with CD8+ or CD163+ score in either cohort, but obesity, compared to normal BMI, was positively associated with CD4+ score (*p* = 0.02) among the expression only cohort. Among the IHC cohort, continuous pre-diagnosis BMI and BMI at age 18 were not associated with any immune score. Among the expression only cohort, continuous pre-diagnosis BMI was positively associated with GSAct (*p* = 0.05), CD8+ (*p* = 0.02), CD4+ (*p* = 0.003), and CD163+ (*p* = 0.03); however, BMI at age 18 was not associated with any immune score.

## Discussion

There is a growing understanding of the critical role of the breast tumor immune microenvironment in treatment response and outcomes in breast cancer, yet modifiable patient factors that impact immune infiltration in breast cancer remain poorly understood. The Nurses’ Health Study offers a large-scale, unique cohort to evaluate interactions between patient lifestyle exposures and the immune microenvironment. Specifically, this study is unique in its integration of multiple immune cell-specific immunohistochemistry (CD8, CD4, CD20, and CD163) with published immune gene expression signatures, including the IRIS gene sets, which we recently demonstrated can effectively interrogate the breast cancer tumor immune microenvironment in a large clinical trial [33]. Using paired IHC and gene expression data from over 250 unique patient tumors for each marker, we successfully derived improved expression-based predictors of immune cell subset infiltration and then evaluated the association with patient factors: BMI change from age 18, physical activity, and EDIP.

Higher BMI change since age 18 demonstrated the strongest association between immune cell-specific expression scores and patient factors, specifically GSAct, CD4 T cell score, and CD163 macrophage score. BMI change in adulthood has been associated with breast cancer risk [34]. The role of obesity in the tumor immune microenvironment is of great interest, driven initially through evidence in melanoma that obesity was associated with longer survival in patients receiving immunotherapy [10]. Mechanistic work suggested that, paradoxically, obesity results in increased immune aging, tumor progression and PD-1-mediated T cell dysfunction yet increased efficacy of PD-1/PD-L1 blockade in murine models and patients with cancer [35]. Several recent papers investigated the association of TILs and BMI. In one, high stromal TILs were associated with increased event-free survival in lean (hazard ratio [HR] = 0.22, 95% CI = 0.08–0.62; *P* = 0.004) but not in heavier patients [36]. A second showed that TIL density was significantly lower in obese than in normal weight and overweight patients [37]. Importantly, neither of these studies evaluated specific immune cell subsets as TILs encompass diverse pro- and anti-tumor immune cells.

While most FDA-approved immunotherapies target CD8+ cytotoxic T cells, the role of CD4+ helper T cells [38, 39] and CD163+ macrophages [40–42] is less well defined but increasingly seen as key players in the breast cancer microenvironment. It is established that T‐lymphocyte populations change with obesity (reviewed in [43]). In our model, for each 10 unit (kg/m^2^) increase in BMI—roughly equivalent to an individual going from BMI 20 (normal weight) to BMI 30 (obese), the percentage of cells positive for CD4 and CD163 increased 1.6% and 1.4%, respectively. It remains unclear whether this percent change could explain differences in response to therapy. In obesity, there is evidence that interferon gamma-producing pro-inflammatory CD4‐positive Th1 cells are increased, whereas anti‐inflammatory CD4‐positive Th2 and Treg cells are reduced [43]. Intriguingly, we have previously shown in the Nurses’ Health Study that higher BMI was associated with increased expression of genes associated with IFN alpha and gamma response in ER- tumor and ER- tumor-adjacent tissues [9]. In addition, in multivariable models of CD8, CD4, CD163, and GeparSixto scores, both HER2 and TNBC status significantly contributed to each model reinforcing the importance of investigating TME metrics within specific breast cancer subtypes.

Inflammatory diet was positively associated with GSAct in bivariable analyses with a trend in multivariable analyses, suggesting that a more pro-inflammatory diet is associated with higher immunity. This differs from our hypothesis, which was based on data in colorectal cancer, where inflammatory diet was associated with a higher risk of developing colorectal cancer *only* among tumors that had low tumor infiltrating lymphocytes [15]. It is likely that the distinct settings, for example, direct exposure of colonic mucosa to dietary elements versus no exposure in breast tissue, may influence these effects. To our knowledge, this is the first study to examine the relationship between inflammatory diet and quantity of TILs and immune subsets in breast tumors.

We also did not find a correlation between physical activity and immune cell-specific expression scores, specifically not GSAct, CD8, or CD4, which had either bivariable or multivariable association with other patient factors. Physical activity has been hypothesized to be associated with biomarkers of inflammation in breast cancer survivors [44]. In animal breast cancer models, effect of physical activity on the amount of TILs is conflicting [45, 46]. In humans, exercise was not found to affect levels of circulating T cells in patients receiving chemotherapy for breast cancer [47]. To our knowledge, this is the first study exploring the effect of physical activity on multiple immune cell subset infiltration in breast tumor tissue and further delineating activity versus inactivity and more granular evaluation of types of physical activity may provide additional insights. While physical activity was not associated with immune cell infiltration in this study, overall, patients with breast cancer have been shown to benefit from exercise during and after cancer-directed therapy, and this study should not be used to justify a sedentary lifestyle for these patients [48, 49].

In this cohort of over 600 tumor–normal pairs and an independent validation cohort (TCGA), both GSAct and *CD8A* expression metrics show modest—but consistent—correlation between breast cancer and tumor-adjacent normal breast tissue. This suggests that the adjacent normal breast may reflect an altered immune microenvironment in the context of breast cancer. In a smaller cohort, inflammation expression was elevated in adjacent normal tissue relative to reduction mammoplasty tissues [50]. In the NHS, elevated inflammatory expression in adjacent normal breast tissues was associated with higher BMI and alcohol consumption specifically in ER- tumors [9, 22]. It is possible that tumor-adjacent normal inflammatory gene expression is a “bystander” effect in response to the tumor. Additional work on the immune microenvironment of tumor-adjacent breast is warranted to understand if adjacent normal immune infiltration is associated with breast carcinogenesis, immune infiltration of established tumors, or therapy response.

This study does have limitations. Only a subset of all the subjects enrolled in NHS/NHSII were included in the TMA due to limited tumor tissue availability. However, the characteristics of participants included in the TMA were very similar to those of all the eligible cases, including BMI, physical activity, EDIP score, and other breast cancer risk factors (e.g., first-degree family history and parity). Adiposity has differential associations with breast cancer risk based on menopausal status, and the models were derived primarily in NHSII subjects and applied primarily in NHS subjects; importantly, menopausal status and NHS cohort were covariates included in multivariable models. We acknowledge that TILs in breast cancer are typically characterized using a standard approach based on the International TILs Working Group [51]; however, given the computer-based quantification approach and diversity around stromal versus epithelial correlation across immune markers, we used total positive cells. In addition, the correlation between novel expression signatures and infiltrating immune cell number was modest, though significantly outperformed multiple established immune cell-specific signatures. We hypothesize that this reflects the fact that bulk transcriptome signatures may not be an optimal way to represent discrete infiltrating immune cells numbers and supports further work on single cell-based technologies such as single cell RNA sequencing and highly multiplexed immunofluorescence profiling.

Future studies of the association of patient factors, specifically BMI and EDIP, and patient outcomes are warranted. It would be interesting to investigate the effect of modification of BMI on patient outcomes with different systemic treatments. Associations between BMI, physical activity, and diet with other immune cell subsets should also be investigated in order to understand the complex relationships between immune cell infiltration and modifiable patient factors. Further, in future studies, the association between immune infiltration signatures and patient prognosis will be assessed, but this requires careful analysis beyond the scope of the present study. In addition, to improve gene expression signatures of specific immune subsets we plan to utilize more complex modeling, such as machine learning; however, a strength of our linear model-based score approach is the ability to identify and quantify contribution of individual components.

## Conclusions

By leveraging a large, unique cohort with classic immune cell marker IHC, gene expression, and patient exposure data, we identify an association of CD4+ and CD163+ immune scores with BMI change since age 18, but not physical activity nor EDIP. With the growing interest in dissecting and targeting the immune microenvironment in breast cancer, these data support further work into the impact of patient exposures and the breast tumor immune microenvironment.

## Supplementary Information

**Additional file 1:** Supplementary Tables S1, S4–S10. **Supplementary Table 1.** Cohort Characteristics; **Supplementary Table 4.** CD8 Immune Signature Lasso Regression Model; **Supplementary Table 5.** CD4 Immune Signature Lasso Regression Model; **Supplementary Table 6.** CD163 Immune Signature Lasso Regression Model; **Supplementary Table 7.** CD20 Immune Signature Lasso Regression Model; **Supplementary Table 8.** Multivariable-adjusted associations of lifestyle factors and immune cell signatures with categorical BMI one-cycle prior to diagnosis; **Supplementary Table 9.** Multivariable-adjusted associations of lifestyle factors and immune cell signatures with continuous BMI one-cycle prior to diagnosis; **Supplementary Table 10.** Multivariable-adjusted associations of lifestyle factors and immune cell signatures with continuous BMI at age 18.

**Additional file 2: Supplementary Table S2.** Immune Gene Expression Scores. Gene expression scores for all samples used in analyses.

**Additional file 3: Supplementary Table S3.** Spearman's correlation of individual signature score with immune marker immunohistochemistry (IHC).

**Additional file 4:** Supplementary Figures S1–S3. **Supplementary Figure 1.** Immune profiling in Nurses’ Health Study. A–B. GeparSixto immune gene expression score by receptor subtype (A) and intrinsic subtype (B). C. CD8 immunohistochemistry (IHC) in epithelial versus stromal compartments, line indicates best fit. D. GeparSixto immune gene expression score versus CD8 IHC epithelial+stromal. E. Correlation of GeparSixto immune gene expression score with CD8A single gene expression. **Supplementary Figure 2.** A–B. Evaluation of only tumors defined as PAM50 normal subtype, evaluating tumor versus normal for GeparSixto immune signature (A) and CD8A single gene expression (B). C–F. GeparSixto immune activation signature (C–D) and CD8A single gene expression (E–F) in tumor and normal blocks by receptor subtype (C, E) and PAM50 intrinsic subtype (D, F). **Supplementary Figure 3.** Correlation matrices of each lasso reduction model versus all other models overall (A), among hormone receptor positive (HR+; B), HER2+ (C), and triplenegative breast cancer (TNBC; D).

## Data Availability

All data relevant to the study are included in the article or uploaded as supplementary information. All gene expression data are available on NCBI Geo, Accession # GSE115577.

## Abbreviations

NHS: Nurses’ Health Study
NHSII: Nurses’ Health Study II
IHC: Immunohistochemistry
BMI: Body mass index
EDIP: Empirical dietary inflammatory pattern
TILs: Tumor-infiltrating lymphocytes
ER: Estrogen receptor
HR: Hormone receptor
PR: Progesterone receptor
HER2: Human epidermal growth factor receptor 2
MET: Metabolic equivalent task
HT: Hormone therapy
TMA: Tumor microarrays
FFPE: Formalin-fixed paraffin-embedded
TCGA: The Cancer Genome Atlas
GSAct: GeparSixto “immune activation signature”
IRIS: Immune response in silico

## References

[1] Denkert C, Loibl S, Noske A, Roller M, Muller BM, Komor M, Budczies J, Darb-Esfahani S, Kronenwett R, Hanusch C (2010). Tumor-associated lymphocytes as an independent predictor of response to neoadjuvant chemotherapy in breast cancer. J Clin Oncol.

[2] Loi S, Sirtaine N, Piette F, Salgado R, Viale G, Van Eenoo F, Rouas G, Francis P, Crown JP, Hitre E (2013). Prognostic and predictive value of tumor-infiltrating lymphocytes in a phase III randomized adjuvant breast cancer trial in node-positive breast cancer comparing the addition of docetaxel to doxorubicin with doxorubicin-based chemotherapy: BIG 02–98. J Clin Oncol.

[3] Mao Y, Qu Q, Zhang Y, Liu J, Chen X, Shen K (2014). The value of tumor infiltrating lymphocytes (TILs) for predicting response to neoadjuvant chemotherapy in breast cancer: a systematic review and meta-analysis. PLoS ONE.

[4] Salgado R, Denkert C, Campbell C, Savas P, Nucifero P, Aura C, de Azambuja E, Eidtmann H, Ellis CE, Baselga J (2015). Tumor-Infiltrating Lymphocytes and Associations With Pathological Complete Response and Event-Free Survival in HER2-Positive Early-Stage Breast Cancer Treated With Lapatinib and Trastuzumab: A Secondary Analysis of the NeoALTTO Trial. JAMA Oncol.

[5] Savas P, Salgado R, Denkert C, Sotiriou C, Darcy PK, Smyth MJ, Loi S (2016). Clinical relevance of host immunity in breast cancer: from TILs to the clinic. Nat Rev Clin Oncol.

[6] Ali HR, Provenzano E, Dawson SJ, Blows FM, Liu B, Shah M, Earl HM, Poole CJ, Hiller L, Dunn JA (2014). Association between CD8+ T-cell infiltration and breast cancer survival in 12,439 patients. Ann Oncol.

[7] Shalapour S, Font-Burgada J, Di Caro G, Zhong Z, Sanchez-Lopez E, Dhar D, Willimsky G, Ammirante M, Strasner A, Hansel DE (2015). Immunosuppressive plasma cells impede T-cell-dependent immunogenic chemotherapy. Nature.

[8] Deng T, Lyon CJ, Bergin S, Caligiuri MA, Hsueh WA (2016). Obesity, Inflammation, and Cancer. Annu Rev Pathol.

[9] Heng YJ, Wang J, Ahearn TU, Brown SB, Zhang X, Ambrosone CB, de Andrade VP, Brufsky AM, Couch FJ, King TA (2019). Molecular mechanisms linking high body mass index to breast cancer etiology in post-menopausal breast tumor and tumor-adjacent tissues. Breast Cancer Res Treat.

[10] McQuade JL, Daniel CR, Hess KR, Mak C, Wang DY, Rai RR, Park JJ, Haydu LE, Spencer C, Wongchenko M (2018). Association of body-mass index and outcomes in patients with metastatic melanoma treated with targeted therapy, immunotherapy, or chemotherapy: a retrospective, multicohort analysis. Lancet Oncol.

[11] Tabung FK, Steck SE, Liese AD, Zhang J, Ma Y, Johnson KC, Lane DS, Qi L, Snetselaar L, Vitolins M, Ockene JK, Hebert JR (2016). Patterns of change over time and history of the inflammatory potential of diet and risk of breast cancer among postmenopausal women. Breast Cancer Res Treat.

[12] Tabung FK (2016). Steck, Susan E, Liese, Angela D, Zhang, Jiajia, Ma, Yunsheng, Caan, Bette, Chlebowski, Rowan T, Freudenheim, Jo L, Hou, Lifang, Mossavar-Rahmani, Yasmin, Shivappa, Nitin, Vitolins, Mara Z, Wactawski-Wende, Jean, Ockene, Judith K, Hebert, James R: Association between dietary inflammatory potential and breast cancer incidence and death: results from the Women's Health Initiative. Br J Cancer.

[13] Tabung FK, Smith-Warner SA, Chavarro JE, Fung TT, Hu FB, Willett WC, Giovannucci EL (2017). An Empirical Dietary Inflammatory Pattern Score Enhances Prediction of Circulating Inflammatory Biomarkers in Adults. J Nutr.

[14] Tabung FK, Smith-Warner SA, Chavarro JE, Wu K, Fuchs CS, Hu FB, Chan AT, Willett WC, Giovannucci EL (2016). Development and Validation of an Empirical Dietary Inflammatory Index. J Nutr.

[15] Liu L, Nishihara R, Qian ZR, Tabung FK, Nevo D, Zhang X, Song M, Cao Y, Mima K, Masugi Y *et al*: Association Between Inflammatory Diet Pattern and Risk of Colorectal Carcinoma Subtypes Classified by Immune Responses to Tumor. *Gastroenterology* 2017, 153(6):1517–1530 e1514. 10.1053/j.gastro.2017.08.045 PMC5705461 28865736

[16] Tamimi RM, Baer HJ, Marotti J, Galan M, Galaburda L, Fu Y, Deitz AC, Connolly JL, Schnitt SJ, Colditz GA (2008). Comparison of molecular phenotypes of ductal carcinoma in situ and invasive breast cancer. Breast Cancer Res.

[17] Kensler KH, Sankar VN, Wang J, Zhang X, Rubadue CA, Baker GM, Parker JS, Hoadley KA, Stancu AL, Pyle ME (2019). PAM50 Molecular Intrinsic Subtypes in the Nurses' Health Study Cohorts. Cancer Epidemiol Biomark Prev.

[18] Troy LM, Hunter DJ, Manson JE, Colditz GA, Stampfer MJ, Willett WC (1995). The validity of recalled weight among younger women. Int J Obes Relat Metab Disord.

[19] Keum N, Greenwood DC, Lee DH, Kim R, Aune D, Ju W, Hu FB, Giovannucci EL: Adult weight gain and adiposity-related cancers: a dose-response meta-analysis of prospective observational studies. *J Natl Cancer Inst* 2015, 107(2). 10.1093/jnci/djv088 25757865

[20] Wolf AM, Hunter DJ, Colditz GA, Manson JE, Stampfer MJ, Corsano KA, Rosner B, Kriska A, Willett WC (1994). Reproducibility and validity of a self-administered physical activity questionnaire. Int J Epidemiol.

[21] McGee EE, Kim CH, Wang M, Spiegelman D, Stover DG, Heng YJ, Collins LC, Baker GM, Farvid MS, Schedin P (2020). Erythrocyte membrane fatty acids and breast cancer risk by tumor tissue expression of immuno-inflammatory markers and fatty acid synthase: a nested case-control study. Breast Cancer Res.

[22] Wang J, Heng YJ, Eliassen AH, Tamimi RM, Hazra A, Carey VJ, Ambrosone CB, de Andrade VP, Brufsky A, Couch FJ (2017). Alcohol consumption and breast tumor gene expression. Breast Cancer Res.

[23] Xu W, Seok J, Mindrinos MN, Schweitzer AC, Jiang H, Wilhelmy J, Clark TA, Kapur K, Xing Y, Faham M (2011). Human transcriptome array for high-throughput clinical studies. Proc Natl Acad Sci.

[24] Atlas TCG (2012). Comprehensive molecular portraits of human breast tumours. Nature.

[25] Stover DG, Coloff JL, Barry WT, Brugge JS, Winer EP, Selfors LM (2016). The Role of Proliferation in Determining Response to Neoadjuvant Chemotherapy in Breast Cancer: A Gene Expression-Based Meta-Analysis. Clin Cancer Res.

[26] Denkert C, von Minckwitz G, Brase JC, Sinn BV, Gade S, Kronenwett R, Pfitzner BM, Salat C, Loi S, Schmitt WD (2015). Tumor-infiltrating lymphocytes and response to neoadjuvant chemotherapy with or without carboplatin in human epidermal growth factor receptor 2-positive and triple-negative primary breast cancers. J Clin Oncol.

[27] Lehmann BD, Bauer JA, Chen X, Sanders ME, Chakravarthy AB, Shyr Y, Pietenpol JA (2011). Identification of human triple-negative breast cancer subtypes and preclinical models for selection of targeted therapies. J Clin Investig.

[28] Desmedt C, Haibe-Kains B, Wirapati P, Buyse M, Larsimont D, Bontempi G, Delorenzi M, Piccart M, Sotiriou C (2008). Biological processes associated with breast cancer clinical outcome depend on the molecular subtypes. Clin Cancer Res.

[29] Rody A, Holtrich U, Pusztai L, Liedtke C, Gaetje R, Ruckhaeberle E, Solbach C, Hanker L, Ahr A, Metzler D (2009). T-cell metagene predicts a favorable prognosis in estrogen receptor-negative and HER2-positive breast cancers. Breast Cancer Res.

[30] Gatza ML, Lucas JE, Barry WT, Kim JW, Wang Q, Crawford MD, Datto MB, Kelley M, Mathey-Prevot B, Potti A (2010). A pathway-based classification of human breast cancer. Proc Natl Acad Sci U S A.

[31] Abbas AR, Baldwin D, Ma Y, Ouyang W, Gurney A, Martin F, Fong S, van Lookeren CM, Godowski P, Williams PM (2005). Immune response in silico (IRIS): immune-specific genes identified from a compendium of microarray expression data. Genes Immun.

[32] Godec J, Tan Y, Liberzon A, Tamayo P, Bhattacharya S, Butte AJ, Mesirov JP, Haining WN (2016). Compendium of Immune Signatures Identifies Conserved and Species-Specific Biology in Response to Inflammation. Immunity.

[33] Filho OM, Stover DG, Asad S, Ansell PJ, Watson M, Loibl S, Geyer CE, Jr., Bae J, Collier K, Cherian M *et al*: Association of Immunophenotype With Pathologic Complete Response to Neoadjuvant Chemotherapy for Triple-Negative Breast Cancer: A Secondary Analysis of the BrighTNess Phase 3 Randomized Clinical Trial. *JAMA oncology* 2021. 10.1001/jamaoncol.2020.7310 PMC7893540 33599688

[34] Gathirua-Mwangi WG, Zollinger TW, Murage MJ, Pradhan KR, Champion VL (2015). Adult BMI change and risk of Breast Cancer: National Health and Nutrition Examination Survey (NHANES) 2005–2010. Breast Cancer.

[35] Wang Z, Aguilar EG, Luna JI, Dunai C, Khuat LT, Le CT, Mirsoian A, Minnar CM, Stoffel KM, Sturgill IR (2019). Paradoxical effects of obesity on T cell function during tumor progression and PD-1 checkpoint blockade. Nat Med.

[36] Floris G, Richard F, Hamy AS, Jongen L, Wildiers H, Ardui J, Punie K, Smeets A, Berteloot P, Vergote I (2021). Body Mass Index and Tumor-Infiltrating Lymphocytes in Triple-Negative Breast Cancer. J Natl Cancer Inst.

[37] Takada K, Kashiwagi S, Asano Y, Goto W, Ishihara S, Morisaki T, Shibutani M, Tanaka H, Hirakawa K, Ohira M (2021). Clinical verification of body mass index and tumor immune response in patients with breast cancer receiving preoperative chemotherapy. BMC Cancer.

[38] Péguillet I, Milder M, Louis D, Vincent-Salomon A, Dorval T, Piperno-Neumann S, Scholl SM, Lantz O (2014). High numbers of differentiated effector CD4 T cells are found in patients with cancer and correlate with clinical response after neoadjuvant therapy of breast cancer. Cancer Res.

[39] Tay RE, Richardson EK, Toh HC (2021). Revisiting the role of CD4(+) T cells in cancer immunotherapy-new insights into old paradigms. Cancer Gene Ther.

[40] Guerriero JL, Sotayo A, Ponichtera HE, Castrillon JA, Pourzia AL, Schad S, Johnson SF, Carrasco RD, Lazo S, Bronson RT (2017). Class IIa HDAC inhibition reduces breast tumours and metastases through anti-tumour macrophages. Nature.

[41] Waks AG, Stover DG, Guerriero JL, Dillon D, Barry WT, Gjini E, Hartl C, Lo W, Savoie J, Brock J (2019). The Immune Microenvironment in Hormone Receptor-Positive Breast Cancer Before and After Preoperative Chemotherapy. Clin Cancer Res.

[42] Li CM, Shapiro H, Tsiobikas C, Selfors LM, Chen H, Rosenbluth J, Moore K, Gupta KP, Gray GK, Oren Y (2020). Aging-Associated Alterations in Mammary Epithelia and Stroma Revealed by Single-Cell RNA Sequencing. Cell Rep.

[43] Picon-Ruiz M, Morata-Tarifa C, Valle-Goffin JJ, Friedman ER, Slingerland JM (2017). Obesity and adverse breast cancer risk and outcome: Mechanistic insights and strategies for intervention. CA Cancer J Clin.

[44] Jones SB, Thomas GA, Hesselsweet SD, Alvarez-Reeves M, Yu H, Irwin ML (2013). Effect of exercise on markers of inflammation in breast cancer survivors: the Yale exercise and survivorship study. Cancer Prev Res (Phila).

[45] Bianco TM, Abdalla DR, Desiderio CS, Thys S, Simoens C, Bogers JP, Murta EFC, Michelin MA (2017). The influence of physical activity in the anti-tumor immune response in experimental breast tumor. Immunol Lett.

[46] Hagar A, Wang Z, Koyama S, Serrano JA, Melo L, Vargas S, Carpenter R, Foley J (2019). Endurance training slows breast tumor growth in mice by suppressing Treg cells recruitment to tumors. BMC Cancer.

[47] Kim JJ, Shin YA, Suk MH (2015). Effect of a 12-week walking exercise program on body composition and immune cell count in patients with breast cancer who are undergoing chemotherapy. J Exerc Nutrition Biochem.

[48] Mijwel S, Jervaeus A, Bolam KA, Norrbom J, Bergh J, Rundqvist H, Wengstrom Y (2019). High-intensity exercise during chemotherapy induces beneficial effects 12 months into breast cancer survivorship. J Cancer Surviv.

[49] Mugele H, Freitag N, Wilhelmi J, Yang Y, Cheng S, Bloch W, Schumann M (2019). High-intensity interval training in the therapy and aftercare of cancer patients: a systematic review with meta-analysis. J Cancer Surviv.

[50] Quigley DA, Tahiri A, Luders T, Riis MH, Balmain A, Borresen-Dale AL, Bukholm I, Kristensen V (2017). Age, estrogen, and immune response in breast adenocarcinoma and adjacent normal tissue. Oncoimmunology.

[51] Salgado R, Denkert C, Demaria S, Sirtaine N, Klauschen F, Pruneri G, Wienert S, Van den Eynden G, Baehner FL, Penault-Llorca F (2015). The evaluation of tumor-infiltrating lymphocytes (TILs) in breast cancer: recommendations by an International TILs Working Group 2014. Ann Oncol.

