# Supplementary material for: Association of body mass index and inflammatory dietary pattern with breast cancer pathologic and genomic immunophenotype in the nurses’ health study
Source: Breast Cancer Res. 2022 Nov 14;24:78. doi: 10.1186/s13058-022-01573-5 (PMC9661734; doi:10.1186/s13058-022-01573-5)
Supplement: Supplementary file 1 — Additional file 1: Supplementary Tables S1, S4–S10. Supplementary Table 1. Cohort Characteristics; Supplementary Table 4. CD8 Immune Signature Lasso Regression Model; Supplementary Table 5. CD4 Immune Signature Lasso Regression Model; Supplementary Table 6. CD163 Immune Signature Lasso Regression Model; Supplementary Table 7. CD20 Immune Signature Lasso Regression Model; Supplementary Table 8. Multivariable-adjusted associations of lifestyle factors and immune cell signatures with categorical BMI one-cycle prior to diagnosis; Supplementary Table 9. Multivariable-adjusted associations of lifestyle factors and immune cell signatures with continuous BMI one-cycle prior to diagnosis; Supplementary Table 10. Multivariable-adjusted associations of lifestyle factors and immune cell signatures with continuous BMI at age 18. [file 13058_2022_1573_MOESM1_ESM.docx]

**Supplementary Tables 1, 4-10**

**Supplementary Table 1. Cohort Characteristics**

|  | **N (%)** |
| --- | --- |
| **Total** | 882 |
| **Immunohistochemistry data** | 262 (29.7) |
| **NHS cohort** |  |
| NHS I | 537 (60.9) |
| NHS II | 345 (39.1) |
| **IHC** **subtype** |  |
| TNBC | 101 (12.4) |
| HR+ | 492 (60.2) |
| HER2+ | 224 (27.4) |
| **Race** |  |
| White | 842 (95.5) |
| Non-White | 40 (4.5) |
| **Diagnosis year** |  |
| Prior to 1990 | 14 (1.6) |
| 1990-1999 | 436 (49.4) |
| 2000-2011 | 432 (49.0) |
| **Menopausal Status**  Premenopausal  Postmenopausal  Unknown | 237 (26.8)  607 (68.8)  38 (4.3) |
| **Stage at diagnosis** |  |
| 1 | 528 (60.0) |
| 2 | 271 (30.8) |
| 3 | 75 (8.5) |
| 4 | 6 (0.7) |
| **Body mass index (BMI)** |  |
| Normal Weight | 440 (50.1) |
| Overweight | 262 (29.8) |
| Obese | 177 (20.1) |
| **Age at diagnosis (years)** |  |
| <50 | 185 (21.0) |
| 50-59 | 269 (30.5) |
| 60-69 | 230 (26.1) |
| >=70 | 198 (22.4) |
| **Cumulative average physical activity (MET hours/week)** |  |
| Mean | 18.4 |
| Standard deviation | 17.2 |
| Range | (0.4,191.3) |
| **Cumulative average empirical**  **dietary inflammatory pattern (EDIP) score** |  |
| Mean | -0.06 |
| Standard deviation | 0.8 |
| Range | (-4.3,4.5) |

**Supplementary Table 4. CD8 Immune Signature Lasso Regression Model**

| **CD8** | Coefficient |
| --- | --- |
| (Intercept) | 10.3477015 |
| GEPARSIXTO.ACT.25534375 | 2.9733223 |
| IFNG.20335537 | -3.0289647 |
| IMMUNE.STAT1.18698033 | 0.6388144 |
| IMMUNESIG1.TNBC.21633166 | 1.4604903 |
| STAT1.19272155 | 2.2601941 |
| GEPARSIXTO.SUPP.25534375 | 1.4773695 |
| MetaImmuneSignature | 2.6374423 |
| GSE22886_NAIVE_VS_MEMORY_TCELL_UP | -2.6294252 |
| GSE22886_NAIVE_CD4_TCELL_VS_NKCELL_UP | -2.8187506 |
| GSE22886_TCELL_VS_BCELL_NAIVE_DN | -0.5784559 |
| GSE22886_CD8_TCELL_VS_BCELL_NAIVE_DN | -0.277566 |
| GSE22886_IGA_VS_IGM_MEMORY_BCELL_UP | -7.0195527 |
| GSE22886_NAIVE_BCELL_VS_BM_PLASMA_CELL_DN | -3.3624872 |
| GSE22886_IGG_IGA_MEMORY_BCELL_VS_BLOOD_PLASMA_CELL_UP | -4.5579612 |
| GSE22886_IGM_MEMORY_BCELL_VS_BM_PLASMA_CELL_DN | -0.243018 |
| GSE22886_DAY0_VS_DAY7_MONOCYTE_IN_CULTURE_DN | -2.8262872 |
| GSE22886_DAY1_VS_DAY7_MONOCYTE_IN_CULTURE_UP | 12.6885009 |
| GSE22886_DC_VS_MONOCYTE_UP | -1.3257099 |
| GSE22886_NAIVE_BCELL_VS_DC_UP | 0.6308482 |
| GSE22886_NAIVE_CD8_TCELL_VS_NEUTROPHIL_DN | -3.8590511 |
| GSE22886_NAIVE_CD8_TCELL_VS_DC_UP | 2.6111184 |
| GSE22886_NAIVE_CD4_TCELL_VS_MONOCYTE_UP | 8.1805989 |
| GSE22886_NAIVE_CD4_TCELL_VS_MONOCYTE_DN | -1.0172186 |
| GSE22886_NAIVE_CD4_TCELL_VS_48H_ACT_TH2_DN | -0.895167 |

**Supplementary Table 5. CD4 Immune Signature Lasso Regression Model**

| **CD4** | Coefficient |
| --- | --- |
| (Intercept) | 13.998198 |
| IFNA.20335537 | -5.3641033 |
| IMMUNE.STAT1.18698033 | 3.294439 |
| IMMUNESIG1.TNBC.21633166 | 3.7123208 |
| IMMUNESIG2.21633166 | 7.8399235 |
| GSE22886_NAIVE_VS_MEMORY_TCELL_UP | -1.1758527 |
| GSE22886_TCELL_VS_BCELL_NAIVE_UP | 4.5008256 |
| GSE22886_TCELL_VS_BCELL_NAIVE_DN | -5.0565217 |
| GSE22886_NAIVE_VS_IGM_MEMORY_BCELL_UP | -3.2207936 |
| GSE22886_NAIVE_BCELL_VS_BLOOD_PLASMA_CELL_UP | -0.6883387 |
| GSE22886_IGG_IGA_MEMORY_BCELL_VS_BLOOD_PLASMA_CELL_DN | -2.7167227 |
| GSE22886_DAY1_VS_DAY7_MONOCYTE_IN_CULTURE_UP | 9.536316 |
| GSE22886_NAIVE_TCELL_VS_MONOCYTE_UP | 0.330514 |
| GSE22886_NAIVE_BCELL_VS_NEUTROPHIL_DN | 1.6479172 |
| GSE22886_NAIVE_CD8_TCELL_VS_NEUTROPHIL_DN | -6.5359211 |
| GSE22886_NAIVE_CD4_TCELL_VS_DC_UP | 0.7216861 |
| GSE22886_NAIVE_CD4_TCELL_VS_MONOCYTE_UP | 3.1570178 |
| GSE22886_TH1_VS_TH2_12H_ACT_UP | -12.41909 |
| GSE22886_NAIVE_CD4_TCELL_VS_48H_ACT_TH2_DN | -0.2922181 |

**Supplementary Table 6. CD163 Immune Signature Lasso Regression Model**

| **CD163** | **Coefficient** |
| --- | --- |
| (Intercept) | 37.1511601 |
| GEPARSIXTO.ACT.25534375 | 4.09500095 |
| IMMUNE.STAT1.18698033 | 5.10544243 |
| GEPARSIXTO.SUPP.25534375 | 2.17259912 |
| GSE22886_NAIVE_VS_MEMORY_TCELL_UP | -4.3921398 |
| GSE22886_CD4_TCELL_VS_BCELL_NAIVE_UP | 0.63847632 |
| GSE22886_NAIVE_VS_IGM_MEMORY_BCELL_UP | -11.841719 |
| GSE22886_IGG_IGA_MEMORY_BCELL_VS_BLOOD_PLASMA_CELL_UP | -12.120949 |
| GSE22886_DAY1_VS_DAY7_MONOCYTE_IN_CULTURE_UP | 9.02437077 |
| GSE22886_NAIVE_BCELL_VS_NEUTROPHIL_DN | 5.0135614 |
| GSE22886_NAIVE_CD8_TCELL_VS_MONOCYTE_DN | 1.64271576 |
| GSE22886_TH1_VS_TH2_12H_ACT_DN | -0.0690807 |
| GSE22886_UNSTIM_VS_IL15_STIM_NKCELL_UP | -6.4226243 |

**Supplementary Table 7. CD20 Immune Signature Lasso Regression Model**

| **CD20** | Coefficient |
| --- | --- |
| (Intercept) | 4.00310294 |
| GEPARSIXTO.ACT.25534375 | 0.47068716 |
| IFNA.20335537 | -0.0931346 |
| IMMUNESIG1.TNBC.21633166 | 2.85209582 |
| IMMUNESIG2.21633166 | 5.61159926 |
| TNFA.20335537 | -0.8269168 |
| GEPARSIXTO.SUPP.25534375 | 0.51513178 |
| GSE22886_CD8_VS_CD4_NAIVE_TCELL_DN | -2.9086578 |
| GSE22886_NAIVE_VS_IGM_MEMORY_BCELL_UP | -10.507907 |
| GSE22886_IGA_VS_IGM_MEMORY_BCELL_DN | -0.6395418 |
| GSE22886_NAIVE_BCELL_VS_BLOOD_PLASMA_CELL_UP | -3.211971 |
| GSE22886_IGM_MEMORY_BCELL_VS_BM_PLASMA_CELL_UP | 2.66332929 |
| GSE22886_DAY0_VS_DAY1_MONOCYTE_IN_CULTURE_DN | -1.6907722 |
| GSE22886_DAY1_VS_DAY7_MONOCYTE_IN_CULTURE_UP | 6.31532803 |
| GSE22886_NAIVE_BCELL_VS_NEUTROPHIL_UP | 1.87351652 |
| GSE22886_NAIVE_BCELL_VS_DC_UP | 4.43697451 |
| GSE22886_NAIVE_BCELL_VS_MONOCYTE_UP | 8.79539996 |
| GSE22886_NAIVE_CD8_TCELL_VS_NEUTROPHIL_DN | -8.4467092 |
| GSE22886_NAIVE_CD4_TCELL_VS_DC_UP | 1.57484458 |
| GSE22886_NAIVE_CD4_TCELL_VS_MONOCYTE_UP | 2.20780092 |
| GSE22886_NAIVE_CD4_TCELL_VS_MONOCYTE_DN | -4.302975 |
| GSE22886_IL2_VS_IL15_STIM_NKCELL_UP | -0.9915478 |
| GSE22886_NAIVE_CD4_TCELL_VS_48H_ACT_TH2_DN | -1.9705099 |
| GSE22886_UNSTIM_VS_IL15_STIM_NKCELL_UP | -0.2138161 |
| GSE22886_UNSTIM_VS_IL15_STIM_NKCELL_DN | -0.514373 |

**Supplementary Table 8. Multivariable-adjusted associations of lifestyle factors and immune cell signatures with categorical BMI one-cycle prior to diagnosis**

|  | | | **IHC Cohort (N=262)** | | | **Expression Only Cohort (N=620)** | | |
| --- | --- | --- | --- | --- | --- | --- | --- | --- |
| **Predictor** | | | **Beta coefficient** | **Standard error** | **p-value** | **Beta coefficient** | **Standard error** | **p-value** |
|  |  | **GeparSixto** | | | | | | |
| *Body Mass Index (BMI)* | | |  |  |  |  |  |  |
| Underweight/Normal (<25) | | | ref | ref | ref | ref | ref | ref |
| Overweight (25-<30) | | | -0.05 | 0.09 | 0.52 | 0.03 | 0.06 | 0.60 |
| Obese (≥30) | | | 0.10 | 0.10 | 0.33 | 0.08 | 0.07 | 0.19 |
| *Cumulative average physical activity (MET hours/wk)* | | | 0.0003 | 0.002 | 0.88 | 0.001 | 0.001 | 0.59 |
| *EDIP score*** | | | 0.06 | 0.05 | 0.24 | 0.04 | 0.03 | 0.22 |
|  |  | **CD8+ Score** | | | | | | |
| *Body Mass Index (BMI)* | | |  |  |  |  |  |  |
| Underweight/Normal (<25) | | | ref | ref | ref | ref | ref | ref |
| Overweight (25-<30) | | | -0.38 | 0.94 | 0.69 | 0.54 | 0.60 | 0.37 |
| Obese (≥30) | | | 0.05 | 1.14 | 0.97 | 1.00 | 0.70 | 0.15 |
| *Cumulative average physical activity (MET hours/wk)* | | | 0.02 | 0.02 | 0.40 | 0.01 | 0.02 | 0.55 |
| *EDIP score*** | | | 0.83 | 0.52 | 0.11 | -0.01 | 0.33 | 0.97 |
|  |  | **CD4+ Score** | | | | | | |
| *Body Mass Index (BMI)* | | |  |  |  |  |  |  |
| Underweight/Normal (<25) | | | ref | ref | ref | ref | ref | ref |
| Overweight (25-<30) | | | -0.49 | 0.97 | 0.61 | 0.81 | 0.62 | 0.20 |
| Obese (≥30) | | | 0.96 | 1.17 | 0.41 | **1.68** | **0.72** | **0.02** |
| *Cumulative average physical activity (MET hours/wk)* | | | 0.02 | 0.02 | 0.31 | 0.01 | 0.02 | 0.71 |
| *EDIP score*** | | | 0.55 | 0.54 | 0.31 | -0.18 | 0.34 | 0.59 |
|  |  | **CD20+ Score** | | | | | | |
| *Body Mass Index (BMI)* | | |  |  |  |  |  |  |
| Underweight/Normal (<25) | | | ref | ref | ref | ref | ref | ref |
| Overweight (25-<30) | | | -0.54 | 0.71 | 0.45 | 0.18 | 0.47 | 0.71 |
| Obese (≥30) | | | -0.50 | 0.85 | 0.56 | 0.27 | 0.54 | 0.62 |
| *Cumulative average physical activity (MET hours/wk)* | | | 0.02 | 0.02 | 0.27 | 0.0002 | 0.01 | 0.99 |
| *EDIP score*** | | | 0.76 | 0.39 | 0.05 | -0.05 | 0.26 | 0.83 |
|  |  | **CD163+ Score** | | | | | | |
| *Body Mass Index (BMI)* | | |  |  |  |  |  |  |
| Underweight/Normal (<25) | | | ref | ref | ref | ref | ref | ref |
| Overweight (25-<30) | | | -0.63 | 1.11 | 0.57 | 0.20 | 0.65 | 0.75 |
| Obese (≥30) | | | 1.00 | 1.34 | 0.46 | 1.10 | 0.76 | 0.15 |
| *Cumulative average physical activity (MET hours/wk)* | | | 0.01 | 0.02 | 0.69 | 0.01 | 0.02 | 0.42 |
| *EDIP score*** | | | 0.50 | 0.61 | 0.42 | 0.14 | 0.36 | 0.70 |

*Multivariable linear regression models were controlled for IHC subtype, race, stage at diagnosis, year at diagnosis, age at diagnosis, NHS Cohort, and menopausal status; **Cumulative average empirical dietary inflammatory pattern (EDIP) score

**Supplementary Table 9. Multivariable-adjusted associations of lifestyle factors and immune cell signatures with continuous BMI one-cycle prior to diagnosis**

|  | | | **IHC Cohort (N=262)** | | | **Expression Only Cohort (N=620)** | | |
| --- | --- | --- | --- | --- | --- | --- | --- | --- |
| **Predictor** | | | **Beta coefficient** | **Standard error** | **p-value** | **Beta coefficient** | **Standard error** | **p-value** |
|  |  | **GeparSixto** | | | | | | |
| *BMI one-cycle prior to diagnosis* | | | 0.01 | 0.01 | 0.23 | **0.01** | **0.01** | **0.05** |
| *Cumulative average physical activity (MET hours/wk)* | | | 0.001 | 0.002 | 0.79 | 0.001 | 0.001 | 0.48 |
| *EDIP score*** | | | 0.06 | 0.05 | 0.20 | 0.03 | 0.03 | 0.33 |
|  |  | **CD8+ Score** | | | | | | |
| *BMI one-cycle prior to diagnosis* | | | 0.05 | 0.08 | 0.53 | **0.13** | **0.06** | **0.02** |
| *Cumulative average physical activity (MET hours/wk)* | | | 0.02 | 0.02 | 0.36 | 0.01 | 0.02 | 0.44 |
| *EDIP score*** | | | 0.80 | 0.51 | 0.11 | -0.11 | 0.33 | 0.73 |
|  |  | **CD4+ Score** | | | | | | |
| *BMI one-cycle prior to diagnosis* | | | 0.08 | 0.08 | 0.32 | **0.17** | **0.06** | **0.003** |
| *Cumulative average physical activity (MET hours/wk)* | | | 0.02 | 0.02 | 0.27 | 0.01 | 0.02 | 0.60 |
| *EDIP score*** | | | 0.58 | 0.53 | 0.27 | -0.27 | 0.34 | 0.43 |
|  |  | **CD20+ Score** | | | | | | |
| *BMI one-cycle prior to diagnosis* | | | -0.01 | 0.06 | 0.91 | 0.07 | 0.04 | 0.12 |
| *Cumulative average physical activity (MET hours/wk)* | | | 0.02 | 0.02 | 0.25 | 0.003 | 0.01 | 0.83 |
| *EDIP score*** | | | 0.72 | 0.38 | 0.06 | -0.14 | 0.26 | 0.59 |
|  |  | **CD163+ Score** | | | | | | |
| *BMI one-cycle prior to diagnosis* | | | 0.07 | 0.09 | 0.42 | **0.13** | **0.06** | **0.03** |
| *Cumulative average physical activity (MET hours/wk)* | | | 0.01 | 0.02 | 0.63 | 0.02 | 0.02 | 0.31 |
| *EDIP score*** | | | 0.54 | 0.60 | 0.37 | 0.04 | 0.36 | 0.91 |

*Multivariable linear regression models were controlled for IHC subtype, race, stage at diagnosis, year at diagnosis, age at diagnosis, NHS Cohort, and menopausal status; **Cumulative average empirical dietary inflammatory pattern (EDIP) score

**Supplementary Table 10. Multivariable-adjusted associations of lifestyle factors and immune cell signatures with continuous BMI at age 18**

|  | | | **IHC Cohort (N=262)** | | | **Expression Only Cohort (N=620)** | | |
| --- | --- | --- | --- | --- | --- | --- | --- | --- |
| **Predictor** | | | **Beta coefficient** | **Standard error** | **p-value** | **Beta coefficient** | **Standard error** | **p-value** |
|  |  | **GeparSixto** | | | | | | |
| *BMI at age 18* | | | 0.002 | 0.01 | 0.88 | 0.002 | 0.01 | 0.81 |
| *Cumulative average physical activity (MET hours/wk)* | | | 0.0003 | 0.002 | 0.86 | 0.001 | 0.001 | 0.73 |
| *EDIP score*** | | | 0.06 | 0.05 | 0.17 | 0.04 | 0.03 | 0.14 |
|  |  | **CD8+ Score** | | | | | | |
| *BMI at age 18* | | | 0.01 | 0.15 | 0.94 | 0.08 | 0.10 | 0.41 |
| *Cumulative average physical activity (MET hours/wk)* | | | 0.02 | 0.02 | 0.39 | 0.01 | 0.02 | 0.76 |
| *EDIP score*** | | | 0.83 | 0.51 | 0.10 | 0.08 | 0.33 | 0.80 |
|  |  | **CD4+ Score** | | | | | | |
| *BMI at age 18* | | | -0.03 | 0.15 | 0.83 | 0.13 | 0.11 | 0.23 |
| *Cumulative average physical activity (MET hours/wk)* | | | 0.02 | 0.02 | 0.30 | 0.004 | 0.02 | 0.83 |
| *EDIP score*** | | | 0.64 | 0.53 | 0.22 | 0.02 | 0.34 | 0.95 |
|  |  | **CD20+ Score** | | | | | | |
| *BMI at age 18* | | | -0.05 | 0.11 | 0.65 | 0.07 | 0.08 | 0.35 |
| *Cumulative average physical activity (MET hours/wk)* | | | 0.02 | 0.02 | 0.24 | -0.003 | 0.01 | 0.84 |
| *EDIP score*** | | | 0.73 | 0.38 | 0.06 | -0.02 | 0.25 | 0.93 |
|  |  | **CD163+ Score** | | | | | | |
| *BMI at age 18* | | | 0.02 | 0.18 | 0.92 | 0.03 | 0.11 | 0.76 |
| *Cumulative average physical activity (MET hours/wk)* | | | 0.01 | 0.02 | 0.67 | 0.01 | 0.02 | 0.53 |
| *EDIP score*** | | | 0.58 | 0.60 | 0.33 | 0.16 | 0.36 | 0.65 |

*Multivariable linear regression models were controlled for IHC subtype, race, stage at diagnosis, year at diagnosis, age at diagnosis, NHS Cohort, and menopausal status; **Cumulative average empirical dietary inflammatory pattern (EDIP) score
