# Supplementary material for: Association of body mass index and inflammatory dietary pattern with breast cancer pathologic and genomic immunophenotype in the nurses’ health study
Source: Breast Cancer Res. 2022 Nov 14;24:78. doi: 10.1186/s13058-022-01573-5 (PMC9661734; doi:10.1186/s13058-022-01573-5)

Supplementary Figure 1

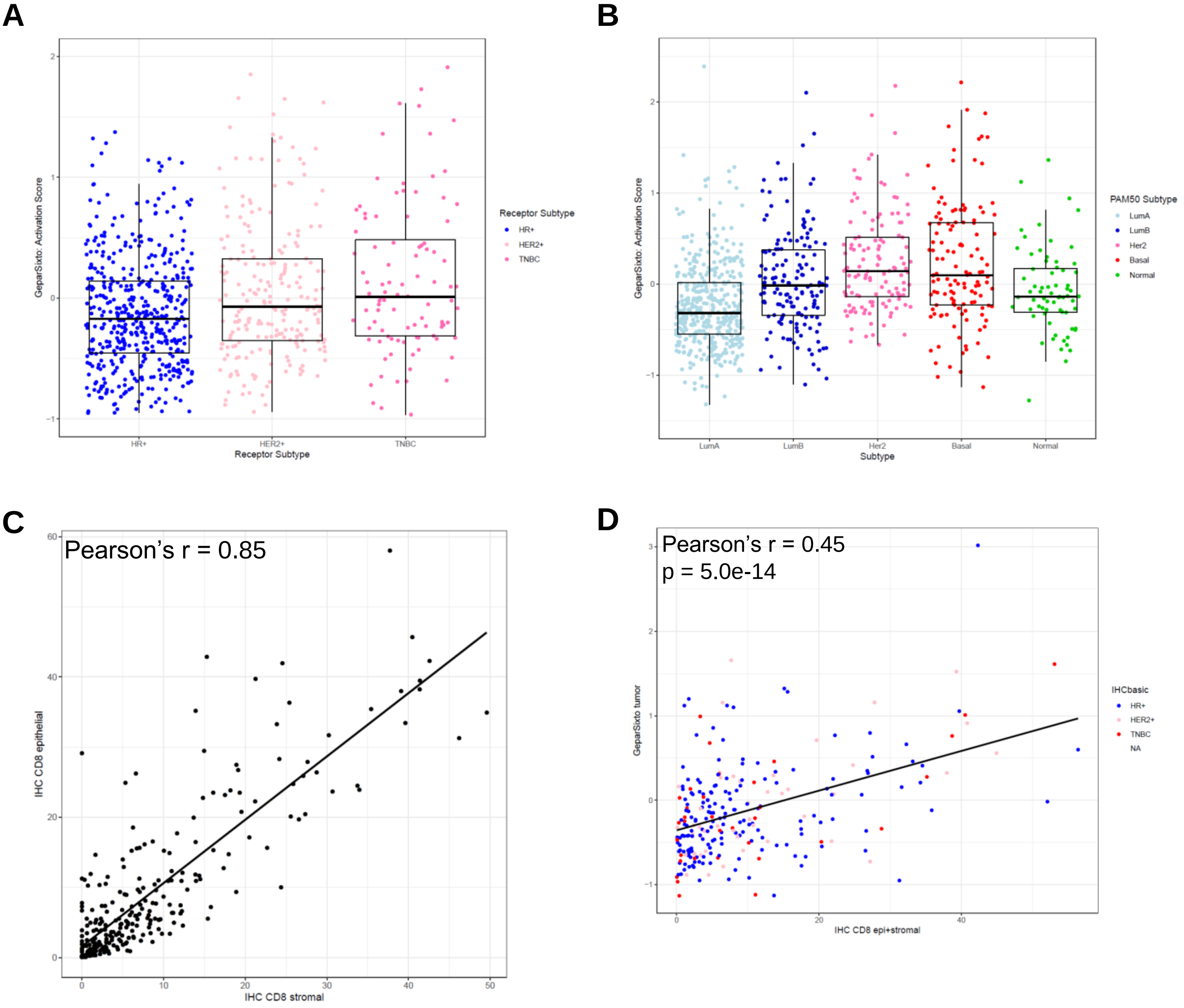

E

Correlation: Pearson's r

	GEPARSIXTO	CD8A Single Gene
CD8 IHC stromal+epithelial	0.453	0.154
CD8 IHC stromal	0.466	0.196
CD8 IHC epithelial	0.456	0.163

Supplementary Figure 2

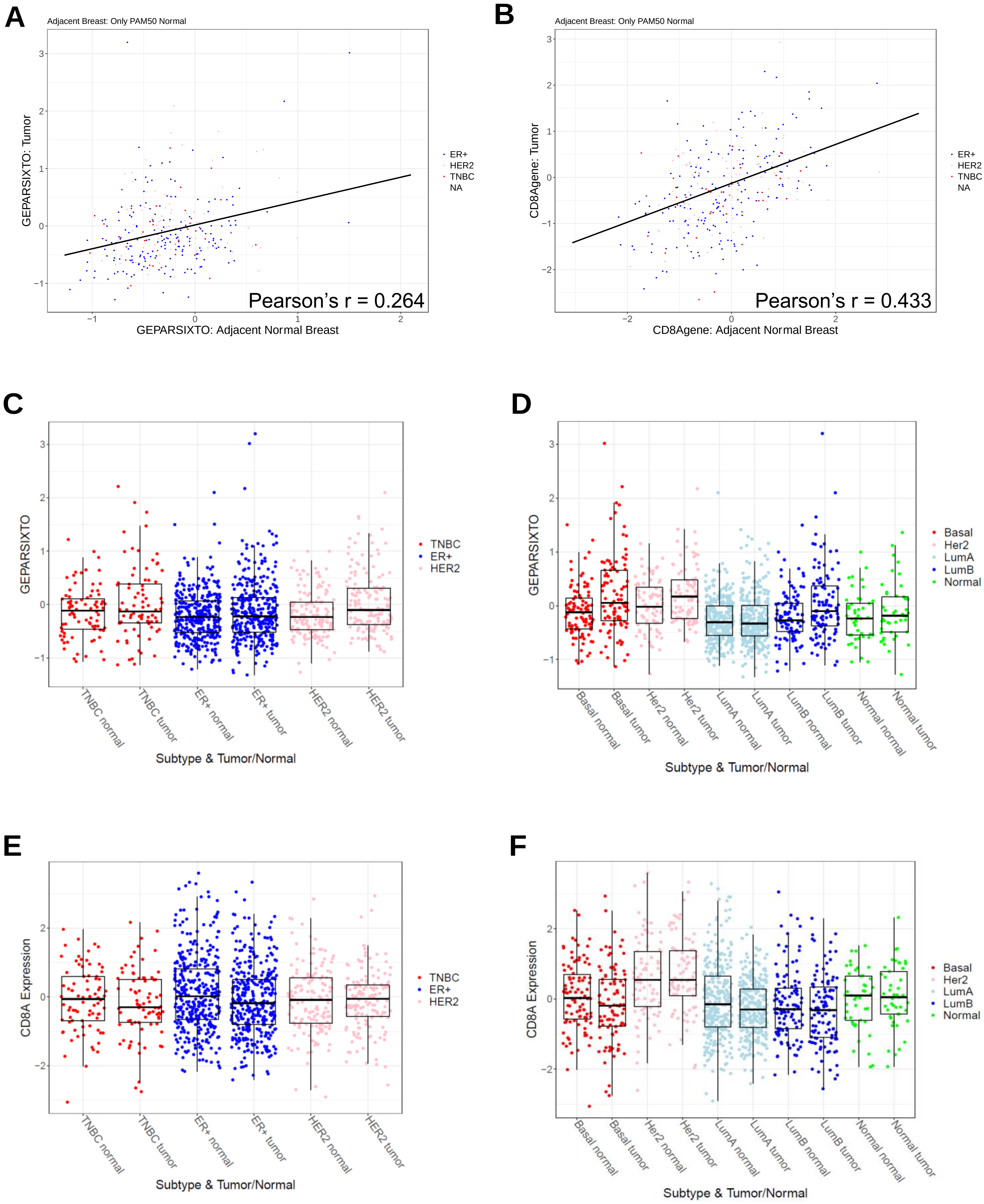

Supplementary Figure 3

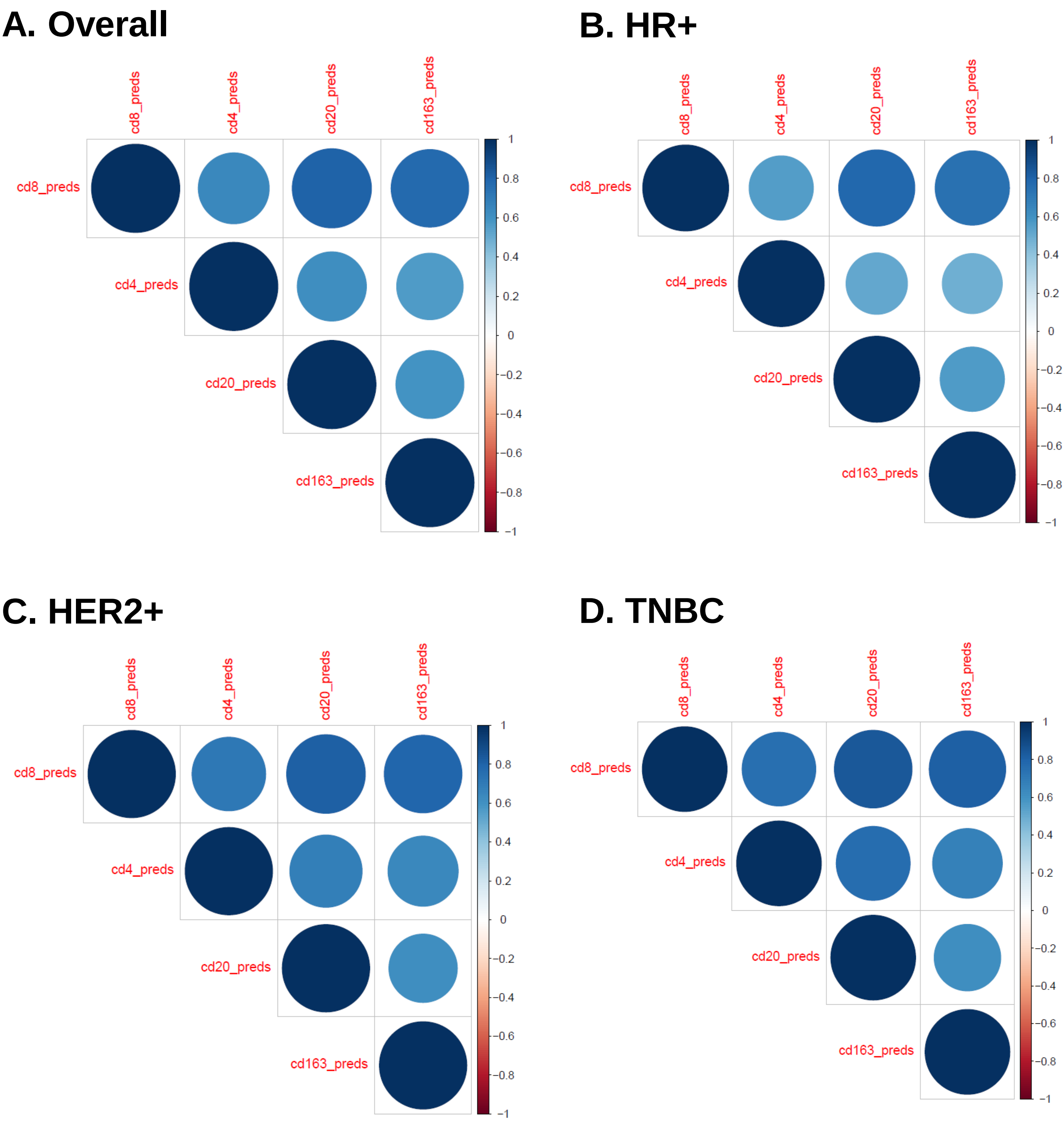

Supplement: Supplementary file 4 — Additional file 4: Supplementary Figures S1–S3. Supplementary Figure 1. Immune profiling in Nurses’ Health Study. A–B. GeparSixto immune gene expression score by receptor subtype (A) and intrinsic subtype (B). C. CD8 immunohistochemistry (IHC) in epithelial versus stromal compartments, line indicates best fit. D. GeparSixto immune gene expression score versus CD8 IHC epithelial+stromal. E. Correlation of GeparSixto immune gene expression score with CD8A single gene expression. Supplementary Figure 2. A–B. Evaluation of only tumors defined as PAM50 normal subtype, evaluating tumor versus normal for GeparSixto immune signature (A) and CD8A single gene expression (B). C–F. GeparSixto immune activation signature (C–D) and CD8A single gene expression (E–F) in tumor and normal blocks by receptor subtype (C, E) and PAM50 intrinsic subtype (D, F). Supplementary Figure 3. Correlation matrices of each lasso reduction model versus all other models overall (A), among hormone receptor positive (HR+; B), HER2+ (C), and triplenegative breast cancer (TNBC; D). [file 13058_2022_1573_MOESM4_ESM.pdf]
